# Light Spectral-Ranged Specific Metabolisms of Plant Pigments

**DOI:** 10.3390/metabo15010001

**Published:** 2024-12-24

**Authors:** The Ngoc Phuong Nguyen, Jwakyung Sung

**Affiliations:** Department of Crop Science, College of Agriculture, Life and Environment Sciences, Chungbuk National University, Cheongju 28644, Republic of Korea; phuongnguyen@chungbuk.ac.kr

**Keywords:** chlorophyll, carotenoid, light spectrum

## Abstract

Chlorophyll is the primary pigment responsible for capturing light energy during photosynthesis, while carotenoids assist in light absorption and provide photoprotection by dissipating excess energy. Both pigments are essential for plant growth and development, playing distinct and complementary roles in maintaining photosynthetic efficiency and protecting plants from oxidative stress. Because of their function in photosynthesis and photoprotection, chlorophyll and carotenoid accumulation are strongly associated with light conditions, especially blue and red lights, which play key roles in regulating their metabolisms. Despite advancements in understanding pigment metabolism, there remains a limited comprehensive overview of how various parts of the light spectrum influence these pathways throughout the entire process. The effects of other spectral ranges of light, such as green light, far-red light, and UV, are not yet fully understood. This review aims to synthesize recent findings about the regulatory network of chlorophyll and carotenoid pathways under different light spectral bands, emphasizing the interplay between light-regulated transcription factors and genes involved in their biosynthesis and degradation.

## 1. Introduction

Chlorophylls are important molecules in photosynthetic processes and are especially responsible for light energy absorption. Chlorophyll absorbs photons in light sources, excites electrons to a higher energy state, and transfers energy into the photosystem, driving the process of photosynthesis. In plants, chlorophyll metabolism is highly coordinated with a series of cooperative reactions and the involvement of various transcription regulators. Chlorophylls are initially biosynthesized from glutamate, then converted to 5-aminolevulinic acid (ALA) and further to protochlorophyllide (Pchlide) by numerous genes such as *HEMA1* (a glutamyl-tRNA reductase protein) and *CHCL* (protoporphyrin IX magnesium chelatase, subunit H) [1]. In plants growing under dark conditions, chlorophyll biosynthesis halts at the step of Pchlide, the immediate precursor of chlorophyllide (Chlide), while light triggers the photoactivity of NADPH-protochlorophyllide oxidoreductase (PORs), catalyzing the conversion of PChlide to Chlide [1,2], and is followed by the chlorophyll cycle to generate chlorophyll a and chlorophyll b. In the degradation process, chlorophyll breaks down into non-toxic compounds, primarily through the actions of enzymes like chlorophyll b reductase (CBR) and pheophorbide a oxygenase (PAO) [3]. Regulation of the levels of chlorophyll and its derivatives is extremely important. Chlorophyll metabolism not only affects the photosynthetic machinery but is also involved in other growth processes such as programmed cell death, the “stay-green” phenomenon, and chloroplast–nucleus communication [4]. Furthermore, over-accumulated Pchlide, a strong photosensitizer, can lead to the generation of reactive oxygen species (ROS), which accelerate growth retardation or cell death [2,4]. Chlorophyll degradation prevents the accumulation of potentially harmful byproducts and is often triggered by stress or aging [3].

Carotenoids are isoprenoid pigments that are essential for plant life. In plants, they provide yellow, orange, and red colors to leaves, flowers, fruits, and some roots [5]. From the MEP pathway, carotenoid biosynthesis begins with the production of precursor molecules like geranylgeranyl diphosphate (GGPP), which undergoes multiple enzyme-driven steps to form carotenoids such as lutein, zeaxanthin, and β-carotene [6]. Key enzymes in this pathway include phytoene synthase (PSY), phytoene desaturase (PDS), and lycopene β-cyclase (LYCB). The biosynthesis process is tightly regulated by transcription factors responsive to light and environmental cues, promoting carotenoid accumulation, particularly under red and blue light [7]. In the context of carotenoid degradation, carotenoids are broken down by carotenoid cleavage dioxygenases (CCDs), converting them into smaller molecules like abscisic acid (ABA) and strigolactones [8]. These degradation products serve as essential molecules in plant growth, stress responses, and signaling. The balance between biosynthesis and degradation ensures that carotenoids support light absorption, photoprotection, and growth regulation while avoiding excess accumulation that could disrupt cellular processes. Apart from providing colors, carotenoids play an important role in protecting photosynthetic apparatus from photooxidative damage [6]. Carotenoids bind with chlorophylls in photosystem (PS) I and PSII and are arranged in the lipid bilayers of the thylakoid membranes, therefore inhibiting the uncontrolled interaction of excited pigments with oxygen that can generate ROS. Additionally, carotenoids act as accessory pigments, absorb light wavelengths that are outside of the chlorophyll absorption range, and provide extra energy for photosynthesis.

**Figure 1:**
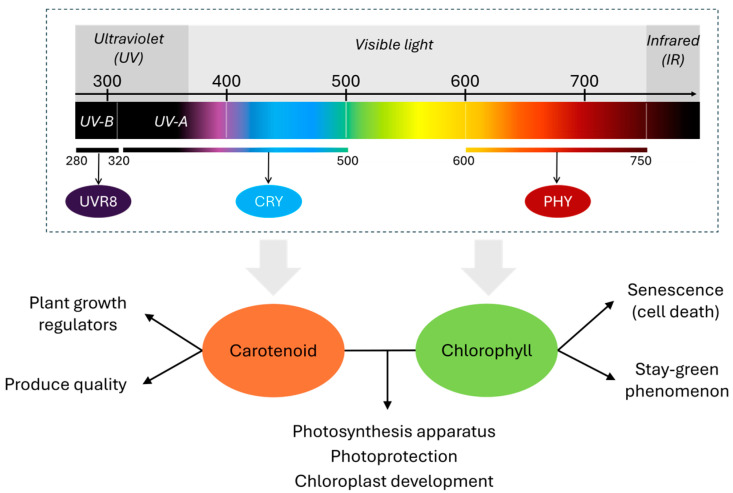
Summary introduction.

Because of their role in photosynthesis and photoprotection, chlorophyll and carotenoid accumulations are strongly associated with light conditions. Light is one of the important factors in controlling the growth and development of plants and the absorption of water and nutrients. The sensing of light in plants is conducted by various light photoreceptors, such as phytochrome (PHY), cryptochrome (CRY), and UV RESISTANCE LOCUS8 (UVR8) [9]. By perceiving light at different compositions, plants can adjust growth through physiological processes such as photosynthesis, accumulation, and transport of photoproducts [10]. Therein, modulating plant pigment metabolism, including chlorophylls and carotenoids, is an essential mechanism to respond to different light spectral ranges (Figure 1). Blue and red lights are deserving of critical attention in understanding the physiological mechanisms of chlorophylls and carotenoids due to the maximum absorption wavelength, thus directly leading to the accumulation of these pigments [11].

Despite recent studies on pigment metabolism, there is a limited comprehensive overview of how the light of different spectral bands influences these pathways, particularly the interaction between transcription factors and key metabolic genes. Especially with carotenoids, many studies reveal a large number of regulators in the transcriptional control of carotenogenic genes [5,12,13,14,15]; however, these results provided fragmented and unassociated insights, and mechanisms and functions of some transcription factors in carotenoid pathways still remain unverified, e.g., transcription factors NAC, MYB [7]. The objective of this review is to share recent knowledge of the effect of different light qualities on chlorophyll and carotenoid pathways, focusing on synthesizing the interplay between light mediate regulators and biosynthesis- and degradation-involved genes.

## 2. Chlorophyll Pathway Under Different Light Spectral Bands

**Figure 2:**
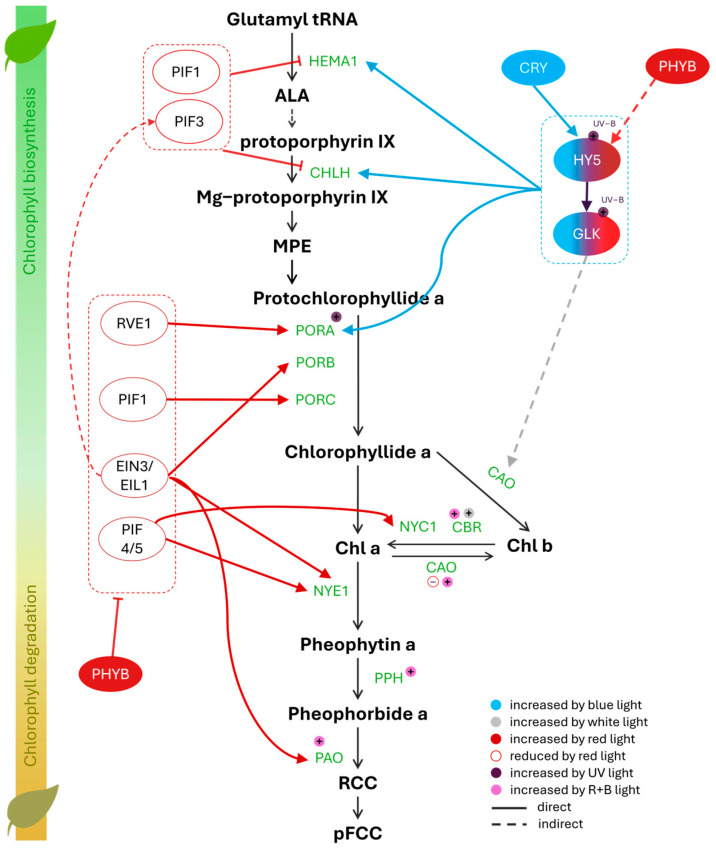
Regulatory network of chlorophyll biosynthesis and degradation pathways under different light spectral ranges. Transcription factors involved are shown in colored oval shapes, with the fill color or border color indicating the positive or negative effect of the corresponding part of the light spectrum.

**Figure 3:**
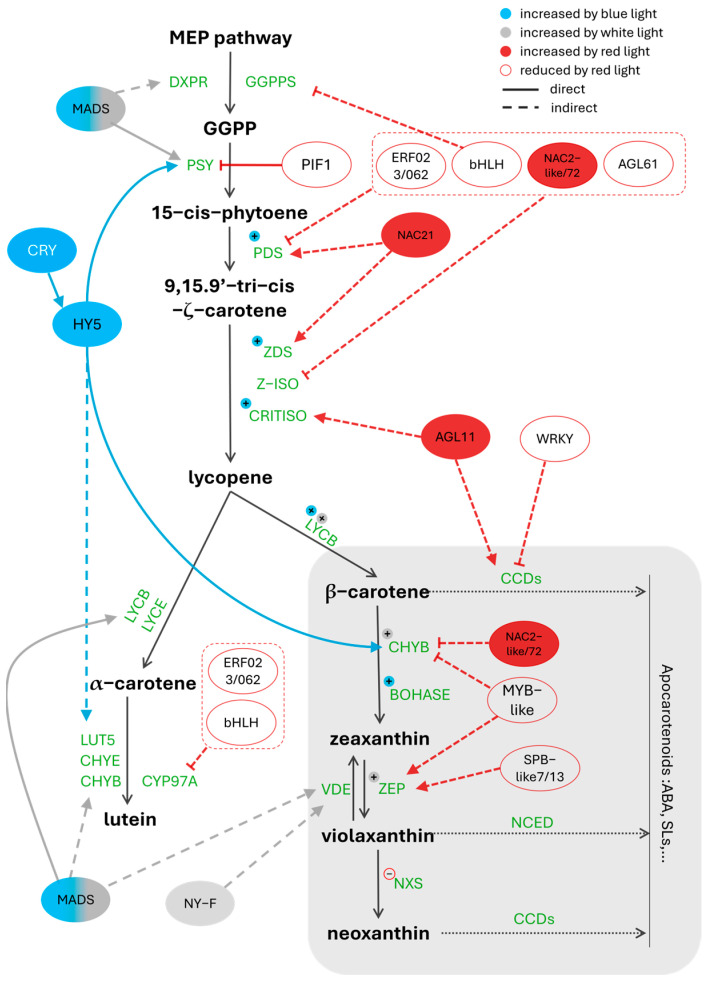
Regulatory network of carotenoid biosynthesis and degradation pathways under different light spectral ranges. Transcription factors involved are shown in colored oval shapes, with the fill color or border color indicating the positive or negative effect of the corresponding part of the light spectrum.

Different light spectral bands were found to be directly involved in the start of the chlorophyll biosynthesis pathway, in which LONG HYPOCOTYL 5 (HY5) and GOLDEN 2-LIKE (GLK) play vital roles in the convergence of blue and red lights (Figure 2). In the dark, the COP1-SPA E3 ligase complex directly enhances HY5 degradation, resulting in a low level of HY5 in plants. With the onset of light, cryptochromes 1/2 (CRY1/2) inhibit the COP1-SPA complex formation and compete with HY5 to COP1 binding, thus increasing the HY5 accumulation [16,17]. HY5 directly binds to the G-box of the GLK promoter and enhances its expression level [18]. Two start genes (*HEMA1*, encoding for glutamyl-tRNA reductase (GluTR) activity, and *CHLH*, responsible for magnesium insertion into the protoporphyrin IX ring in the chlorophyll branch) are both positively enhanced by HY5 and GLK [19,20]. On the other hand, monochromatic red light shows a more complex effect on chlorophyll metabolism through the activities of phytochrome B (PHYB), a red light receptor (Figure 3). PHYA/B is also involved in disrupting the formation of the COP1-SPA complex, thereby regulating the activity of HY5 [16]. Furthermore, PHYB is an inactive repressor of light responses such as phytochrome-interacting factor 1 and 3 (PIF1, PIF3), which depress either *HEMA1* or *CHLH*, thus partly inducing the start of chlorophyll biosynthesis [21,22]. However, in the later pathway, red light acts as a negative regulator for the chlorophyll cycle. Three NADPH-protochlorophyllide oxidoreductase (*POR*) genes, *PORA*, *PORB,* and *PORC*, induce the conversion of Pchlide to Chlide under light conditions but are downregulated by red light through the inhibiting activity of their positive regulators such as RVE1, PIF1, and EIN3/EIL1 [23,24,25]. PHYB takes the role as a repressor of EIN3/EIL1 and PIF4/5, resulting in depressing chlorophyll reduction genes like *NYC1*, *NYE1*, and *PAO* [3,26]. Interestingly, the combination of red and blue light significantly increases the expression levels of genes involved in the chlorophyll cycle and degradation such as *CAO, CBR,* and *PAO* (with a ratio of red to blue light at 3:1) and *PPH* (at a 1:1 ratio) [20]. By contrast, white light significantly boosts the expression level of *CBR* compared with other red/blue combinations [20], suggesting a possible role for green light in regulating downstream genes. In plants, green light can be absorbed by the lower leaves in a complex capony, involved in plant responses to shade, which causes the increased synthesis of chlorophyll *b* [27,28]. However, the effect of green light on chlorophyll pathways remains unclear and requires further investigation.

Recent research suggests that UV-B (280–315 nm) light also upregulates the expression of upstream genes. In tea leaves, UV-B irradiation enhances the activity of CsGLK1/2, directly leading to higher levels of its target genes *CsHEMA1*, *CsCHLH*, and *CsPORA* [29]. Although the expression of CSGLK1/2 decreases significantly under UV-A (315–400 nm), that of *CsPORA* increases under both UV-A and UV-B lights, thus revealing the possible existence of other regulators involved in UV light signal transduction pathways that are responsible for controlling *CsPORA* expression.

## 3. Carotenoid Pathway Under Different Light Spectral Bands

Red light is positively involved in regulating carotenoid accumulation through a complicated interplay between various transcription factors and carotenoid biosynthesis- and cleavage-related genes (Figure 3). Transcriptome analysis reveals that transcription factors from different families, such as bHLH, NAC, MYB AGL, ERF, WRKY, SPB, and PIF, directly or indirectly modulate the expression of carotenogenic genes, including *GGPPS2*, *PDS*, *Z-ISO*, *ZDS2/7*, *CRTISO3, CYP97A*, *CHYB*, *ZEP2*, and *CCD1-2* under red light treatment [12,30]. Red light downregulates some transcription factors that are negatively correlated with carotenoid biosynthesis genes, such as bHLH128 as a *GGPPS*- and *Z-ISO*-depressor, ERF023/062 and AGL61 as *PDS*- and *Z-ISO*-depressors, MYB-like and SBPlike-7/13 as *ZEP*-depressors [14,30]. In addition, PHYB inhibits PIF1, which directly targets the promoter of the phytoene synthase gene (*PSY*) and reduces its expression level [31,32]. In other ways, red light enhances positive regulators like NAC21 and AGL11, both induced the lycopene formation [33]. Carotenoid cleavage dioxygenases (CCDs), which catalyze carotenoid breakdowns to apocarotenoids, are found to be regulated by WRKY transcription factors in *Osmanthus fragrans* [34]. Under red light treatment, WRKY is downregulated in the ripening grapefruit, which is accompanied by the reduction of CCD1-2 transcription expression.

Compared with red light and dark conditions, blue and white light are reported to significantly increase total carotenoid accumulation, with the greatest enhancements of lutein and β-carotene concentrations [12]. Both these light qualities upregulate the activity of HY5 and MADS, which are positively active upstream genes like DXPR and PSY [5,32,35]. HY5 can directly bind to the CHYB promoter and increase its expression. HY5 and MADS also are reported to be positively correlated with the increased expression of key carotenoid biosynthesis genes, including *LYCB*, *LYCE*, *LUT5*, *CHYB*, and *βOHASE*. MAD and HY5 can directly bind to LYCB and CHYB promoters, respectively, and increase their expression [36]. In maize sprouts, blue light promotes the upregulation of *PDS*, *ZDS*, and *CRITISO*, while white light significantly upregulates genes like *CHXB*, *ZEP*, and *VDE* through the activity of MADS and NY-F compared with red or blue light only [12], leading to higher levels of lutein, zeaxanthin, and β-carotene.

## 4. Conclusions and Perspective

Overall, chlorophyll and carotenoid metabolisms are strongly influenced by light spectra, with blue and red lights playing key roles in regulating their biosynthesis and degradation. Blue light promotes chlorophyll accumulation, while the effect of red light varies with its combination with blue light. The combination of red and blue lights is recognized as enhancing an abundance in carotenoid. The red/blue light combination has long been known to increase chlorophyll and carotenoid levels in many plants, but the underlying mechanisms are not fully understood. In this review, we propose a regulatory network that could explain why plants benefit from this combination. This network can contribute to predicting and regulating plant responses to different types of light. By understanding these effects, light composition can be fine-tuned for each plant species, optimizing the light systems to meet growers’ goals. Understanding the interplay between light spectrum and plant pigments has transformative applications in agriculture production. Chlorophyll and carotenoids play vital roles in maintaining photosynthetic efficiency and protecting plants from oxidative stress, and are indirectly involved in other light-induced processes. With the use of artificial lighting as a supplement or alternative to natural light, farmers nowadays can tailor their lighting system to enhance specific light spectrums, such as blue or UV light. Therefore, modulating chlorophyll and carotenoid pathways through a light regime could enable precise control over plant development and stress tolerance, thus promoting crop yield and quality.

These insights also support the enhancement of nutritional and esthetic qualities in crops during the cultivation and and storage period. For instance, in vegetable leaves, blue light irradiation can increase the carotenoid content, especially lutein and β-carotene, which provide vitamin A and act as antioxidants in the human body [6]. Post-harvest applications include using specific light treatments to stabilize pigments and maintain color, flavor, and nutritional value during storage and transport [37,38].

## Data Availability

Not applicable.
